# Feasibility and acceptability of a rural, pragmatic, telemedicine‐delivered healthy lifestyle programme

**DOI:** 10.1002/osp4.366

**Published:** 2019-10-17

**Authors:** John A. Batsis, Auden C. McClure, Aaron B. Weintraub, David F. Kotz, Sivan Rotenberg, Summer B. Cook, Diane Gilbert‐Diamond, Kevin Curtis, Courtney J. Stevens, Diane Sette, Richard I. Rothstein

**Affiliations:** ^1^ Section of General Internal Medicine Dartmouth‐Hitchcock Medical Center Lebanon New Hampshire; ^2^ Geisel School of Medicine at Dartmouth and The Dartmouth Institute for Health Policy and Clinical Practice Lebanon New Hampshire; ^3^ Dartmouth Centers for Health and Aging Dartmouth College Hanover New Hampshire; ^4^ Health Promotion Research Center at Dartmouth Lebanon New Hampshire; ^5^ Section of Weight & Wellness, Department of Medicine Dartmouth‐Hitchcock Lebanon New Hampshire; ^6^ Department of Pediatrics Dartmouth‐Hitchcock Lebanon New Hampshire; ^7^ Department of Computer Science Dartmouth College Hanover New Hampshire; ^8^ Department of Psychiatry Dartmouth‐Hitchcock Lebanon New Hampshire; ^9^ University of New Hampshire Durham New Hampshire; ^10^ Department of Epidemiology Geisel School of Medicine at Dartmouth Hanover New Hampshire; ^11^ Section of Emergency Medicine Dartmouth‐Hitchcock Lebanon New Hampshire

**Keywords:** obesity, pragmatic, rural, telemedicine

## Abstract

**Background:**

The public health crisis of obesity leads to increasing morbidity that are even more profound in certain populations such as rural adults. Live, two‐way video‐conferencing is a modality that can potentially surmount geographic barriers and staffing shortages.

**Methods:**

Patients from the Dartmouth‐Hitchcock Weight and Wellness Center were recruited into a pragmatic, single‐arm, nonrandomized study of a remotely delivered 16‐week evidence‐based healthy lifestyle programme. Patients were provided hardware and appropriate software allowing for remote participation in all sessions, outside of the clinic setting. Our primary outcomes were feasibility and acceptability of the telemedicine intervention, as well as potential effectiveness on anthropometric and functional measures.

**Results:**

Of 62 participants approached, we enrolled 37, of which 27 completed at least 75% of the 16‐week programme sessions (27% attrition). Mean age was 46.9 ± 11.6 years (88.9% female), with a mean body mass index of 41.3 ± 7.1 kg/m^2^ and mean waist circumference of 120.7 ± 16.8 cm. Mean patient participant satisfaction regarding the telemedicine approach was favourable (4.48 ± 0.58 on 1‐5 Likert scale—low to high) and 67.6/75 on standardized questionnaire. Mean weight loss at 16 weeks was 2.22 ± 3.18 kg representing a 2.1% change (*P* < .001), with a loss in waist circumference of 3.4% (*P* = .001). Fat mass and visceral fat were significantly lower at 16 weeks (2.9% and 12.5%; both *P* < .05), with marginal improvement in appendicular skeletal muscle mass (1.7%). In the 30‐second sit‐to‐stand test, a mean improvement of 2.46 stands (*P* = .005) was observed.

**Conclusion:**

A telemedicine‐delivered, intensive weight loss intervention is feasible, acceptable, and potentially effective in rural adults seeking weight loss.

## 1 INTRODUCTION

As a major public health crisis nationally and internationally, obesity rates continue to rise, exceeding^1^ an estimated 38%. Obesity is known to adversely impact cardiometabolic factors, including hypertension, diabetes, and dyslipidemia,^2^ ultimately increasing vascular risk and leading to disability^3^ and death.^4^ The escalating costs associated with obesity in the United States demonstrate a critical need to address this epidemic^5^ given that direct and indirect costs account for 9.3% of the gross domestic product.^6^ Conditions are even worse in rural areas of the United States where obesity prevalence rates are much higher^7^ and patients often need to travel extensive distances to access health care services and specialist providers.^8^, ^9^, ^10^, ^11^ Disparities in accessing care are especially problematic in caring for patients with obesity, where regular interactions are needed to promote health behaviour change.^12^

Mobile health interventions hold promise in engaging adults with obesity in behavioural change. For instance, self‐monitoring using commercial applications has demonstrated an increased likelihood of short‐term weight loss.^13^, ^14^ Goal setting through text messaging,^15^ automated voice response systems,^16^ or tailored self‐monitoring platforms^17^ can all enhance success and are cost‐effective strategies to at‐risk populations. However, engagement drops off after initial usage suggesting a need for more personalized approaches.^18^ In fact, at 12 months, there are no differences in weight loss between digital and control arms.^19^ Recent studies using just‐in‐time adaptive interventions also hold considerable promise in influencing patterns of behaviour for engaging and sustaining weight loss.^20^

The emergence of telemedicine, two‐way live video‐conferencing, has been embraced by the Centers for Medicare and Medicaid services^21^ as a different type of mobile health modality that can potentially surmount geographic barriers to health care delivery. With the advent of policy changes promoting rural broadband and cellular access,^22^ telemedicine is increasingly available to both health care entities and patients alike. In rural obesity management, telemedicine is particularly promising because it reduces demands on patients' time by reducing the need to travel long distances and spend hours away from work^23^ in order to attend high‐intensity visits recommended by the 2013 guidelines.^12^ While an initial investment is needed, the payoff is significant in that it may reduce costs.^24^ The affordability can allow rural patients increased access to specialists, making it a plausible method to deliver care.

Few trials have evaluated the use of telemedicine in obesity management. The Veterans Affairs MOVE! trial has implemented telemedicine in effective and sustainable approaches.^25^, ^26^ Their programme, although, focused only on veterans with obesity across the United States and was not specific to rural areas. The delivery was based on using a telehealth monitor delivering electronic modules, rather than using a clinical care provider. Other studies focus on paediatric populations with hybrid models (in‐person and remote),^27^, ^28^, ^29^, ^30^ or the potential efficacy of low‐intensity models.^31^, ^32^ Studies have demonstrated mixed results in other populations, including pregnancy^33^ or endometrial cancer survivors.^34^ While diet‐quality and obesity are strongly associated with rural health care resource use,^35^ there is a lack of pragmatic research strategies for delivering high‐intensity obesity therapy in rural areas. Our hypothesis in this pilot study was that an adaptation of an in‐person, 16‐week intensive lifestyle intervention could feasibly be delivered using telemedicine and would be acceptable and potentially effective for participants.

## 2 METHODS

### 2.1 Study design and setting

This was a single‐arm, non‐randomized study by enrolling participants attending the Dartmouth‐Hitchcock (D‐H) Weight and Wellness Center between November 2017 and September 2018. D‐H is a 396‐bed hospital serving over 1.5 million persons in the region and situated in Lebanon, NH, on the New Hampshire and Vermont border in Grafton County, which is classified as rural according to the 2010 census.^36^ Sixty five percent of persons live in a health professional shortage area or medically underserved area. The centre was initiated in 2016 and, at that time, evaluated 385 new consultations for adult obesity management yearly. At the time of the study, staffing consisted of three physicians, an advanced practice registered nurse, a behavioural psychologist, a registered nurse exercise specialist, two health coaches, two registered dietitians, and administrative staff. Outcomes were assessed at an in‐person baseline and 16‐week visit. The study was approved by the Committee for the Protection of Human Subjects at Dartmouth College and NCT03309787.

### 2.2 Intervention description

The Healthy Lifestyle Program consisted of a 16‐week programme delivered by a health coach, registered dietitian, and nurse exercise specialist (see Table 1) focused on health‐behaviour change and based on the Diabetes Prevention Program.^37^ Medication management and bariatric surgery are separate programmes within the clinic. Participants are referred from their primary care physicians and complete an initial comprehensive multidisciplinary intake prior to entering the lifestyle programme. As part of the lifestyle programme, patients have the option of choosing in‐person individual or group (up to 15‐20) formats for weekly coaching visits. For the pilot, participants evaluated in the clinic were offered the opportunity to complete 30‐minute individual 1:1 coaching visits remotely via video‐conferencing (see below) after the initial evaluation, in lieu of in‐person care. Other participants were eligible for bariatric surgery or medication management and did not enter this programme. The structure and operational infrastructure paralleled that observed within on‐site care. In addition, participants were provided with a wearable fitness device during the study to enable them to track their physical activity as part of a separate research study.

**Table 1:** Components of the Healthy Lifestyle Program at the Dartmouth Weight and Wellness Center

Staff	Week	Content
Health coach	Week 1^a^	Mindfulness, goal setting
	Week 2	Hunger awareness, mindful eating
	Week 3	Working with emotions
Exercise trainer	Week 4	Movement vs exercise
Health coach	Week 5	Managing thoughts
	Week 6	Stress + social support
	Week 7	Problem solving
Exercise trainer	Week 8	Myths and truths
Registered dietitian	Week 9	Detoxing your diet and food tracking
	Week 10	Food label reading and serving size
	Week 11	Meal planning, grocery shopping, preparing for success
Exercise trainer	Week 12	Sorting through the noise
Registered dietitian	Week 13	The power of protein
	Week 14	Healthy carbohydrates
	Week 15	Good/bad fats, review of the toolbox
Exercise trainer	Week 16	Moving forward

Week 1 occurs after the initial visit at the center.

### 2.3 Telemedicine delivery

The D‐H Center for Telehealth has an extensive infrastructure to support clinical initiatives within D‐H and provided logistical and technical support for this project. All study staff (health coaches, nurse, registered dietitians) were participated in multiple, on‐site training sessions to ensure familiarity with the telehealth platform. Live, mock sessions and ongoing on‐site support was provided by the research assistant (RA) and by a technology consultant from the Center for Telehealth. All communications were conducted through an HIPAA‐compliant *Vidyo* software platform that includes end‐to‐end data encryption using HTTPS (browsing), TLS (signalling), and AES encryption. Coaching sessions were conducted in a private clinical space, using a T450s Lenovo laptop and Logitech H390 USB Headset with a noise‐cancelling microphone. Participants were provided with a Samsung Galaxy Tab A 10.1 tablet that was encrypted per institutional policies to conduct the intervention off‐site (ie, home) with the same software allowing them to interact with the study personnel.

### 2.4 Recruitment and enrolment

Clinic schedules were initially reviewed by the RA. New patients were approached by the clinician and introduced the study opportunity to assess interest. The RA then further described the study, obtained consent, and scheduled a 1‐hour individual orientation for all subjects. Inclusion criteria consisted of English‐speaking, community‐dwelling adults, aged 18 to 65 years with a body mass index (BMI) ≥ 30 kg/m^2^ and otherwise willing to participate in the Healthy Lifestyle Program if recommended by the clinical provider. An additional requirement was access to high‐speed internet access with Wi‐Fi. All patients required an electronic medical record patient portal account; if one was not available, the RA assisted in its creation. Participants were excluded if they were unwilling to participate, as well as those with a medical record diagnosis of dementia, life‐threatening illness, psychiatric illness (untreated serious mental illness, suicidal ideation) precluding their participation in the study, or a history of bariatric surgery. All participants required medical clearance from their primary care provider and needed to provide voluntary written consent. The lead author (JAB) was responsible for training the RA during this process. All participants received a $20 incentive at each in‐person outcome assessment.

### 2.5 Outcomes

Baseline measures were chosen a priori based on their validity, brevity, use in routine clinical care, and availability in the electronic medical record. The RA obtained baseline demographic information and co‐morbid health conditions from the EMR. On‐site assessments occurred at baseline and at 16 weeks, with additional surveys conducted at 4‐week intervals (data not shown). Monthly weights were acquired using an A&D Medical Bluetooth enabled scale and captured using the application. Surveys were sent electronically using REDCap, a secure, web‐based application designed to support research data capture.^38^

Height was measured using a wall‐mounted stadiometer (SECA 216, Hamburg, Germany), with the participant standing barefoot, against a wall, with their weight evenly distributed on both feet and heels together. Three height measurements were taken, and the average was used as the final value. Waist circumference was measured by a registered nurse using a tape measure placed around the abdomen, just above the iliac crest, snug, and not compressing the skin. The participant was asked to relax, and measurements were taken at end‐exhalation. A bioelectrical impedance analyser (SECA mBCA 514, Hamburg, Germany) was used to assess weight, body fat, muscle mass, and visceral fat. Participants were instructed to remove any outer clothing, jewelry, shoes and socks, or tights and stood on the metal electrodes on the base of the machine, facing forward. A 6‐minute walk test was conducted by a nurse according to standard protocols,^39^ in a 100‐ft hallway; a clinically significant difference was defined as 50 m.^40^, ^41^ Last, a 30‐second sit‐to‐stand test^42^ was conducted, administered using an armless chair placed against a wall. Participants were instructed to sit in the middle of the chair, with their backs straight and feet shoulder width apart, with their arms crossed at the wrists and held against their chest. The participant was encouraged to complete as many repetitions of being fully seated to standing within the 30 seconds. Participants also completed the Yip Telemedicine Scale^43^ that has been validated in patients with diabetes at the 16‐week timepoint only; it is a 15‐item, 5‐point scale (maximum score 75) representing satisfaction with telemedicine delivered interventions. Additional acceptability questionnaires were asked at the conclusion of the study. An exit‐interview at the end of the study was conducted that ascertained the participant's impressions of the overall programme and what they liked/disliked about the programme. These interviews were digitally audio‐recorded and transcribed by http://www.rev.com, a commercial transcription programme.

### 2.6 Analysis

All data were aggregated into REDCap. Descriptive statistics (means, standard deviations, medians, proportions, and range) were computed to assess feasibility and acceptability. The analysis focused on completers of the programme. Change in weight, percent weight loss, and waist circumference were our primary preliminary effectiveness outcomes. Paired *t* tests assessed change between baseline and follow‐up. All qualitative interview data were inputted into *Dedoose* and analysed by two researchers using thematic data analysis^44^, ^45^, ^46^ consisting of “open coding” of transcripts, a process of labelling text to identify concepts related to acceptability.^47^ This process enhances rigor by allowing for different views.^48^ Codes were determined both a priori and inductively derived. Text excerpts were aggregated by code to distill patterns and themes related to the intervention's acceptability. The analysis was conducted using STATA v.15, Microsoft Excel 2017, and REDCap's data output for simple quantitative data analyses. While a *P* value < .05 was considered statistically significant, this pilot study was intended to investigate feasibility and was not powered to detect a statistically significant difference in our outcomes.

## 3 RESULTS

### 3.1 Feasibility of recruitment and retention

**Figure 1:**
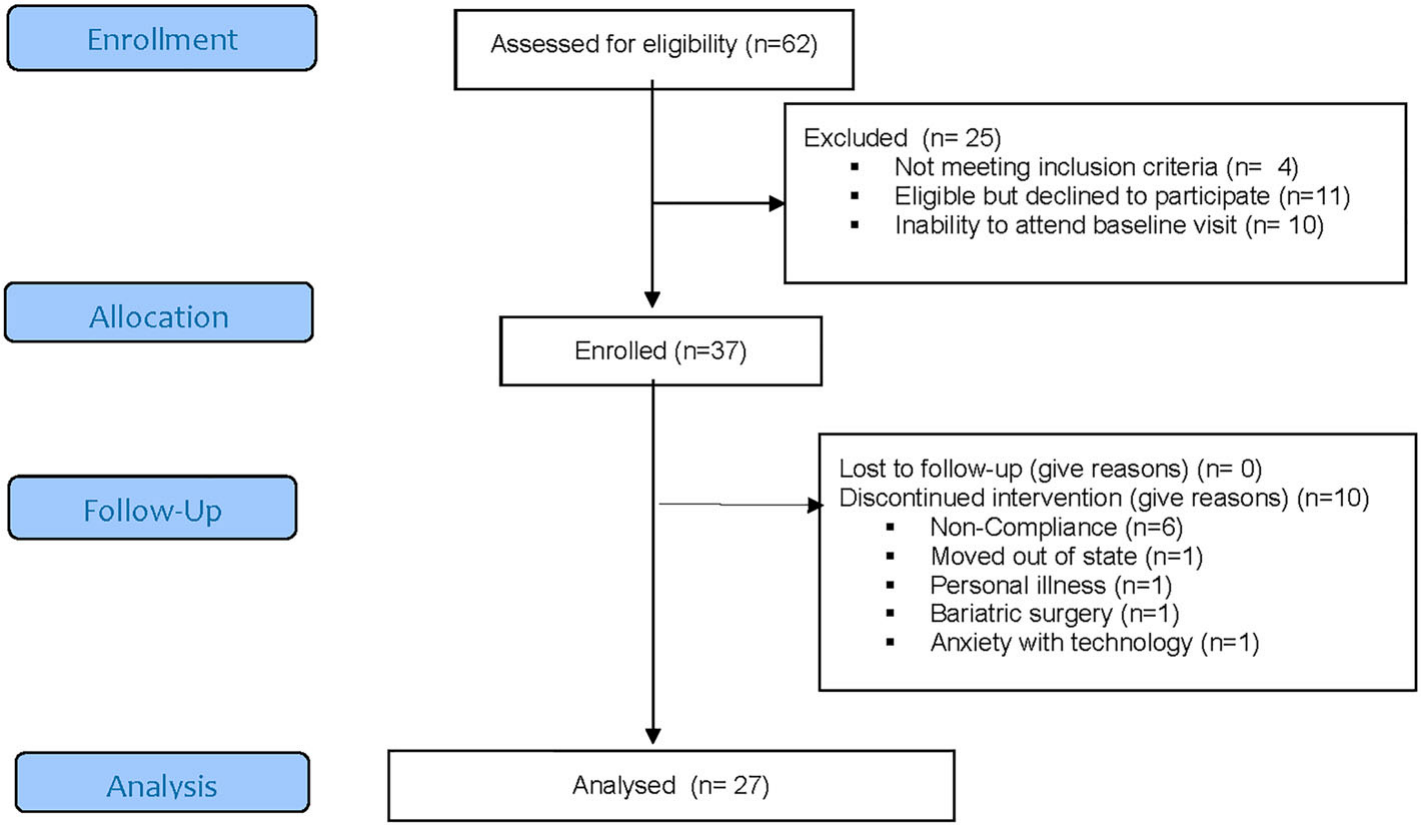
Consort diagram of all participants using telemedicine in a rural, academic, and obesity clinic

Clinicians approached 62 participants seeking treatment at the centre (Figure 1) of which 58 were eligible (93.5%) based on screening demographics. There were 37 participants enrolled (63.8%) exceeding our target of 30 patients. Of the 21/58 that were eligible but declined participation, 11 (19.0%) were unable to participate due to timing/logistical reasons, and 10 were uninterested participate in a video‐delivered intervention (17.2%). A total of 27 participants of the 37 enrolled (75.7%) completed the study; a successful attrition rate was defined as less than 20%. The most common reason for study discontinuation was patient participant noncompliance despite attempted communications to reach them; the clinic's policy is to shift patients to MD directed care if they missed three visits in the lifestyle programme. Only one participant voiced that their discontinuation was due to issues pertaining to the technology.

### 3.2 Intervention adherence

All participants completed all study measures at baseline and follow‐up points while enrolled, exceeding the a priori threshold of 80% considered as successful. The proportion of study participants completing greater than 75% of sessions was favourable among those enrolled (73%) and among those completing the study (100%). Approximately 93%, 96%, and 67% of participants attended greater than 75% of health coach, nurse, and dietitian sessions, respectively.

### 3.3 Baseline characteristics

Cohort characteristics, both enrolled and completers, are presented in Table 2. There were no significant differences between completers and noncompleters except for insurance status. Mean age among those completing the study was 46.9 ± 11.6 (range 27‐64 years), and the proportion of females was high (88.9%). All participants represented themselves as white and not Hispanic. Mean body mass index was 41.3 ± 7.1 kg/m^2^ and mean waist circumference was 120.7 ± 16.8 cm.

**Table 2:** Baseline characteristics of participants

	Overall	Completers	Noncompleters	*P* Value
	N = 37	n = 27	n = 10	
Age, years	46.9 ± 11.6	46.1 ± 12.3	48.9 ± 9.8	.52
Range, years	27‐64	27‐64	27‐60	
Female sex (%)	32 (86.5)	24 (88.9)	8 (80.0)	.48
Race, n (%)				
White	37 (100)	27 (100)	10 (100)	
Hispanic status	0 (0)	0 (0)	0 (0)	
Primary insurance, n (%)				
Medicare	4 (10.8)	2 (7.4)	2 (20.0)	.28
Medicaid	6 (16.2)	2 (7.4)	4 (40.0)	.02
Private	28 (75.7)	23 (85.2)	5 (50.0)	.03
Self‐Pay	‐	‐	‐	
Smoking status, n (%)				
Current	1 (2.7)	1 (3.7)	‐	
Former	12 (32.4)	7 (25.9)	5 (50.0)	.17
Never	24 (64.9)	19 (70.4)	5 (50.0)	.25
Weight, kg	116.5 ± 28.8	113.1 ± 25.4	125.7 ± 36.5	.24
BMI, kg/m^2^				
Range	31.8‐79.9	31.8‐56.5	35.3‐79.9	
Mean	42.2 ± 9.1	41.3 ± 7.1	44.7 ± 13.3	.32
Median	38.8 (36.2, 47.2)	38.8 (36.0, 45.6)	39.5 (37.2, 47.0)	
Waist circumference, cm				
Mean	122.2 ± 19.1	120.7 ± 16.8	126.0 ± 24.6	.46
Range	99.1‐185.0	99.1‐161.0	102.1‐185.0	
Comorbidities, n (%)				
Anxiety	10 (32.3)	8 (34.8)	2 (25.0)	.58
Cognitive impairment	‐	‐	‐	‐
COPD	‐	‐	‐	‐
CAD	‐	‐	‐	‐
Depression	18 (58.1)	13 (56.5)	5 (62.5)	.75
Diabetes	7 (22.6)	6 (26.1)	1 (12.5)	.38
Fibromyalgia	2 (6.5)	1 (4.3)	1 (12.5)	.38
High cholesterol	2 (6.5)	2 (8.7)	‐	
Hypertension	10 (32.3)	9 (39.1)	1 (12.5)	.13
Nonskin cancer	‐	‐	‐	
NAFLD	6 (19.4)	6 (26.1)	‐	
Osteoarthritis	3 (9.7)	1 (4.3)	2 (25.0)	.06
Rheumatologic disease	3 (9.7)	3 (13.0)	‐	
OSA	12 (38.7)	7 (30.4)	5 (62.5)	.08
Stroke	1 (3.2)	1 (4.3)	‐	

*Note*. All variables indicated are represented as mean ± standard deviations, or counts (%).

Abbreviations: BMI, body mass index; COPD, chronic obstructive pulmonary disease; CAD, coronary artery disease; NAFLD, nonalcoholic fatty liver disease; OSA, obstructive sleep apnoea.

### 3.4 Participant acceptability of telemedicine

**Figure 2:**
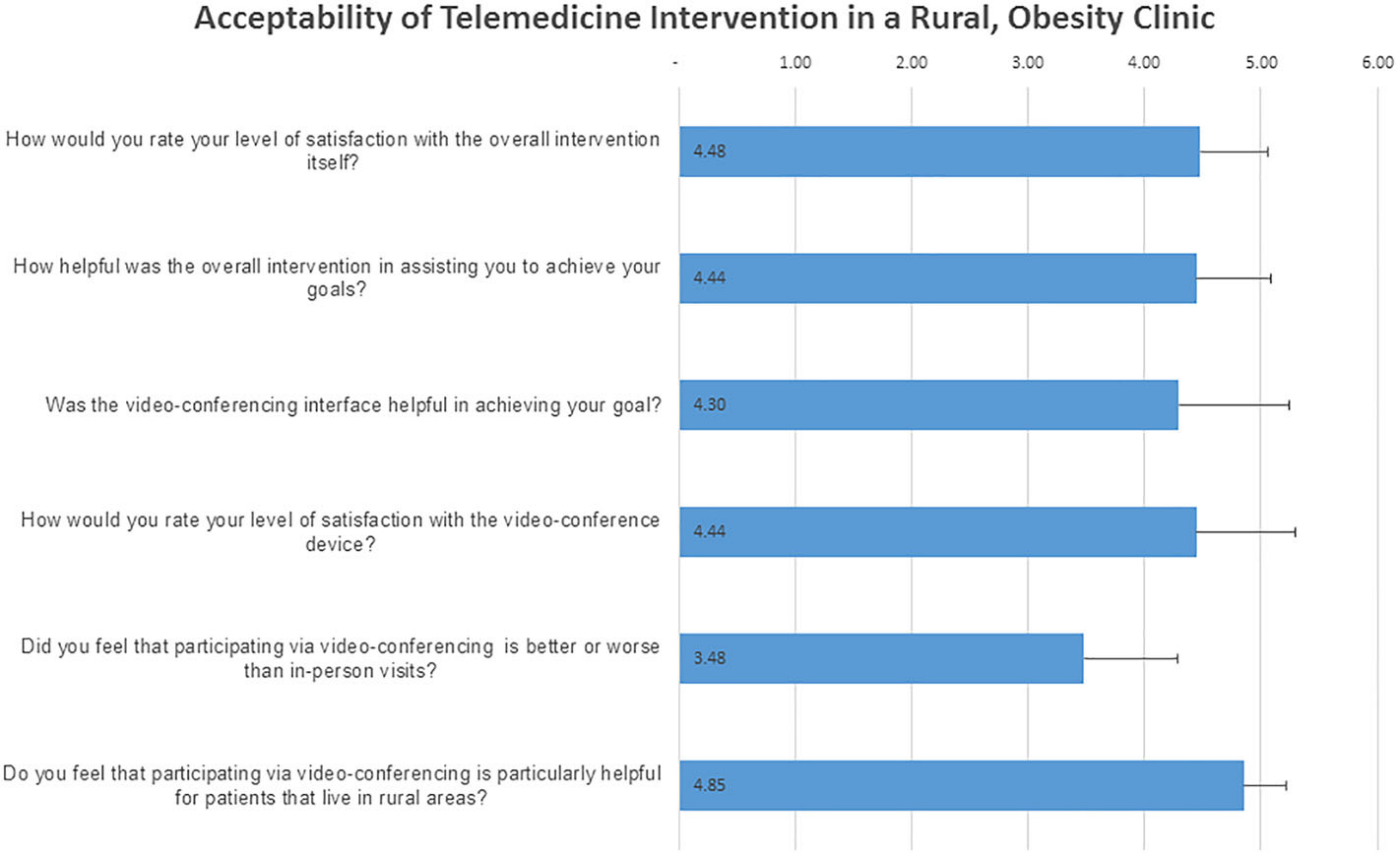
Select questions asked to participants on the acceptability of the intervention. Each question was rated from strongly disagree/dissatisfied (1) to strongly agree/satisfied (5). Mean scores are indicated with error bars representing standard deviations

Figure 2 presents data on the acceptability of telemedicine as a delivery modality. All responses were favourable (Table S2). Specifically, the mean level of satisfaction with the overall intervention was 4.48 ± 0.58 (median 5; range 3‐5), and individuals reported that the programme helped them to achieve their goals (4.44 ± 0.64, 5; range 3‐5). Overall, 92.6% (n = 25/27) of completers would have recommended the intervention to their family/friends. Video‐conferencing was considered an acceptable modality in allowing individuals to achieve their goals (mean 4.30 ± 0.95, median 5, range 1‐5). The Yip Telemedicine questionnaire (Table S1), a marker of telemedicine satisfaction, also suggested that the delivery modality was favourable to participants (mean 67.6 ± 6.95 range 53‐75). The staff did not experience any software or technical issues. Of the 430 sessions, 15 (3.5%) were delayed and only three (0.7%) were cancelled due to technical issues that included bandwidth issues or that a tablet was not charged.

### 3.5 Qualitative inquiry on the programme's acceptability

Many themes emerged through our participant end‐of‐study interviews (Table 3). The importance of time‐savings was observed throughout many of the conversations. Participants were highly positive about video‐conferencing rather than commuting for an in‐person evaluation. This enhanced control of their time, reduced anxiety and hassles, notably in enabling, and allowed for health consultations to occur within the context of a demanding job. Another theme included the simplicity of the video‐conferencing technology. The information delivery was helpful to all participants, and the programme provided considerable resources to enhance nutritional and behavioural strategies. In contrast, a significant criticism was the lack of peer‐support by participants and that a programme wholly based on video‐conferencing felt depersonalizing.

**Table 3:** Representative quotes highlighting acceptability

Theme	Representative Quotation
Delivery remotely vs in‐person	You can be in your pajamas if you want to and do it [telemedicine]
	[I can] live my natural life, without little to no disruption
Time‐savings	Time. There's no commute … You can do it in less than 24 hours, as long as it's set up on this side. It can be very flexible. There's no charge for gas, there's none of that stuff, which is fantastic.
	Definitely the saving on my time and my travel, because I live in Vermont, at least two hours away, two and a half hours away, and I have to leave work half a day at least to get here in order to be here on time before you guys are done for the day.
Simplicity of use	User interface [is really] quite simple
	Oh, this was by far the easiest, the most user‐friendly [intervention]; I feel like I can do this on my own.
Lack of face‐to‐face	I felt very much like an island, like I'm out there struggling all by myself and I can't do it.
	A face group where people who are doing the same thing can communicate
	You need more face‐to‐face, in‐person, and groups type things that you get via telemedicine.

### 3.6 Preliminary effectiveness

Table 4 demonstrates the preliminary effectiveness outcomes of the 27 completers. Over the 16‐week study period, completers lost 2.22 ± 3.18 kg, representing a 2.1% change (*P* < .001) from baseline to follow‐up. Of the completers, 19% lost more than 5% of their weight and waist circumference dropped 3.4% (−4.1 ± 5.9 cm; *P* = .001). Body composition measures were all significantly different at follow‐up (*P* < .05), with reductions in fat mass (2.9%), visceral fat (12.5%), and marginal improvements in appendicular skeletal muscle mass (1.7%). There were improvements (*P* = .005) in the 30‐second sit‐to‐stand test (39% with an improvement of 2) but no significant changes in 6‐minute walk test (*P* = .23).

**Table 4:** Effectiveness outcomes (completers n = 27)

	Baseline	Follow‐up	Delta^a^	% Change	Range	*P* Value
Weight^a^, kg	113.07 ± 25.4	110.8 ± 25.8	2.22 ± 3.18	−2.1 ± 3.0	−3.8, 9.6	<.001
Body mass index, kg/m^2^	41.3 ± 7.1	40.5 ± 7.3	−0.88 ±1.2	−2.1 ± 3.0%	−1.65, 3.43	<.001
Waist circumference^b^, cm	120.6 ± 16.7	116.5 ± 17.0	−4.1 ± 5.9	−3.4 ± 5.0	−16.0, 8.0	.001
% pre/post Δ WC	‐	‐			+7.5, −14.6	‐
Body composition						
Fat mass, %	49.2 ± 6.0	47.9 ± 6.6	−1.33 ± 1.13	−2.87 ± 2.53	−3.8, 1.0	<.001
Muscle mass, kg/height^2^	50.8 ± 6.0	52.1 ± 6.6	1.33 ± 1.13	+2.55 ± 2.17	−1, 3.8	<.001
ASM, kg	15.4 ± 3.35	15.7 ± 3.6	0.26 ± 0.57	1.7 ± 4.0%	−3.7, 15.1	.03
Visceral fat, L	5.08 ± 3.16	4.48 ± 2.79	−0.60 ± 0.86	−12.4 ± 17.5	−2.8, 1.0	.001
6‐minute walk test, m	466.6 ± 105.4	484.6 ± 98.8	18.0 ± 59.5	4.9 ± 12.9	−16.7, 30.7	.55
% with >50 m improvement			7 (29.2)			
30s sit‐to‐stand, # stands	16.2 ± 4.96	19.1 ± 7.3	2.46 ± 3.90	14.2 ± 20.1	−18.2, 50	.005

a Delta represents only data on full data (baseline, follow‐up) of completers.

b Missing data in n = 4 STS and n = 3 6mwt.

## 4 DISCUSSION

An evidence‐based weight loss intervention delivered using telemedicine was feasible and acceptable to rural adults with obesity. Importantly, the intervention led not only to weight loss but also to significant changes in visceral fat as measured by bioelectrical impedance with maintenance in appendicular muscle mass and improvements in strength measures. These results suggest that a future intervention using this delivery modality within a clinical setting can potentially overcome many hurdles/barriers to delivering high‐quality, intensive obesity care in this rural population.

Many previously published obesity interventions occur within primary care environments^49^ or in research centres.^50^ Interventions such as the diabetes prevention programme are effective,^51^ but their reach and dissemination, particularly in rural areas, are limited.^52^ The importance of novel delivery methods such as telemedicine is that it can overcome geographic and operational barriers that have previously impeded the delivery of evidence‐based interventions widely. Among other pragmatic obesity trials in the literature, the majority of obesity trials using telemedicine have focused on paediatric,^27^, ^30^, ^53^, ^54^ workplace,^55^ or veterans affairs populations.^26^, ^56^ Others have used telemedicine in disparity populations for weight maintenance.^57^ To our knowledge, this telemedicine‐delivered multicomponent intervention is the first delivered from a rural, tertiary care medical centre that provides specialty obesity care. Findings from this pilot project demonstrate that participants felt positively about video‐conferencing and that telemedicine could be effective and feasible in obesity management programmes in rural settings.

While adherence to the intervention, as represented by attendance and completion of outcome assessments, was high, the programme suffers from similar engagement issues that plague other obesity programmes, both in research and clinical settings. The clinical programme was specifically designed so that the first 8 weeks are health‐coach intensive and the last 8 weeks are predominantly dietitian related. While weight loss was observed, only 19% lost more than 5% of their body weight. This success rate is partially attributed to many factors. First, most comprehensive weight loss interventions last a minimum of 6 months rather than 16 weeks and such weight loss is represented in this period of time; long‐term outcomes and maintenance are needed. Second, while behaviours are critical to long‐standing behavioural change, a caloric reduction is the key component to losing weight. The educational materials and tools related to nutrition were presented in the latter parts of the intervention; hence, the extent of weight loss that would have expected if this information was delivered earlier in the study period may not have been observed. Future studies could alter the order of the sessions. Third, it is unclear whether the telemedicine modality impacted weight loss and if a hybrid (in‐person and remote) model is needed to augment and enhance weight loss that take advantage of peer and group leader relationships for support. The attrition rate parallels those observed in other studies**.** The study population's readiness to change, ascertained through qualitative inquiry, which is known to impact willingness to engage in health promotion programmes, may have also played a factor. Marginal improvements in appendicular skeletal muscle mass could also account for improvements in function. During weight loss efforts, not only fat is lost but also muscle^58^; it is also possible that the nurse‐led resistance exercise sessions may have had a positive impact on body composition. These results suggest that further testing of the dose‐dependence of exercise training during weight loss interventions is warranted for this middle‐aged adult population within a clinical setting.

Such pilot findings should be interpreted with caution as the design was not randomized. The staff was also limited in space and resources. The evaluated intervention lasted only 16 weeks rather than the recommended minimum of 6 months and 14 encounters for high‐intensity obesity interventions.^12^ Strengths include the team's ability to recruit within the centre, the completion of outcome assessments by participants, and the acceptability of telemedicine to rural adults. It is unknown whether telemedicine may be as acceptable to urban‐dwelling populations, other rural populations, or parts of the country with ready access to services in delivering obesity care. Such an at‐risk, rural population with obesity at risk for health disparities would not ordinarily have access to these services.

## 5 CONCLUSION

A multicomponent, telemedicine‐based obesity lifestyle programme appears to be feasible and acceptable to patients and is thus a promising approach for weight and visceral fat loss in rural populations. A randomized controlled trial is needed to evaluate this modality for future implementation and effectiveness as part of their routine practice.

## CONFLICT OF INTEREST

There are no conflicts of interest pertaining to this manuscript.

## AUTHOR CONTRIBUTIONS

J.A.B., A.C.M., A.B.W., and D.G.D. analysed and interpreted the data. J.A.B., A.C.M., D.F.K., S.R., S.B.C., D.G.D., and R.I.R. were involved in conceiving the study design. All authors read and approved the final manuscript and provided critical input in the revision of the manuscript.

## FUNDING

Dr Batsis receives funding from the National Institute on Aging of the National Institutes of Health under Award Number K23AG051681 and from the Friends of the Norris Cotton Cancer Center at Dartmouth and National Cancer Institute Cancer Center Support Grant 5P30 CA023108‐37 Developmental Funds. Dr Batsis also receives funding from the Patient Centered Oriented Research Institute. Dr Batsis has also received honoraria from the Royal College of Physicians of Ireland, Endocrine Society, and Dinse, Knapp, McAndrew LLC, legal firm. Support was also provided by the Department of Medicine and the Dartmouth Health Promotion and Disease Prevention Research Center supported by Cooperative Agreement Number U48DP005018 from the Centers for Disease Control and Prevention. The Dartmouth Clinical and Translational Science Institute, under award number UL1TR001086 from the National Center for Advancing Translational Sciences (NCATS) of the National Institutes of Health (NIH).

## Supporting information


Table S1. Telemedicine Satisfaction Questionnaire
Table S2. Acceptability of Telemedicine Intervention
