# Supplementary material for: Feasibility and acceptability of a rural, pragmatic, telemedicine‐delivered healthy lifestyle programme
Source: Obes Sci Pract. 2019 Oct 17;5(6):521–30. doi: 10.1002/osp4.366 (PMC6934425; doi:10.1002/osp4.366)
Supplement: Supplementary file 1 — Table S1. Telemedicine Satisfaction Questionnaire Table S2. Acceptability of Telemedicine Intervention [file OSP4-5-521-s001.docx]

**Supplemental Table 1 – Telemedicine Satisfaction Questionnaire**

**Yip Telemedicine Questionnaire:**

|  | **Mean ± SD** | **Range** |
| --- | --- | --- |
| 1. I can see my health-care provider as if we met in person | 4.67±0.55 | 3-5 |
| 1. Telemedicine saves me time traveling to hospital or a specialist clinic | 4.89±0.32 | 4-5 |
| 1. Overall, I am satisfied with the quality of service being provided via telemedicine | 4.67±0.48 | 4-5 |
| 1. I can clearly hear my health-care provider | 4.67±0.55 | 3-5 |
| 1. I think the health-care provided by use of telemedicine is consistent | 4.67±0.55 | 3-5 |
| 1. Telemedicine provides for my health-care needs | 4.48±0.70 | 3-5 |
| 1. I find telemedicine an acceptable way to receive health-care services | 4.41±0.80 | 3-5 |
| 1. I obtain better access to health-care services by use of telemedicine | 3.48±0.94 | 2-5 |
| 1. I feel comfortable communicating with my health-care provider | 4.63±0.49 | 4-5 |
| 1. I do receive adequate attention | 4.63±0.56 | 3-5 |
| 1. I can easily talk to my health-care provider | 4.59±0.64 | 3-5 |
| 1. The health-care provider is able to understand my health-care condition | 4.63±0.56 | 3-5 |
| 1. I meet with the health-care provider more frequently via telemedicine | 4.19±1.08 | 1-5 |
| 1. I do not need assistance while using the system | 4.48±0.85 | 2-5 |
| 1. I will use telemedicine services again | 4.52±0.70 | 3-5 |
|  |  |  |
| **TOTAL SCORE (75 points maximum)** | 67.6 ± 6.95 | 53.0-75.0 |

In their original paper, each of the questions were score 1-5 and then a mean ± standard deviation for a total score of 75.

Abbreviations: SD – standard deviation

**Supplemental Table 2: Acceptability of Telemedicine Intervention**

| **Question** | **Mean** | **Median** | **Range** |
| --- | --- | --- | --- |
| How would you rate your level of satisfaction with the overall intervention Itself? | 4.48±0.58 | 5 | 3-5 |
| How helpful was the overall intervention in assisting you to achieve your goals? | 4.44±0.64 | 5 | 3-5 |
| Was the video-conferencing interface helpful in achieving your goal | 4.30±0.95 | 5 | 1-5 |
| How would you rate your level of satisfaction with the number of: |  |  |  |
| Health coaching sessions (n=6) | 4.41±0.97 | 5 | 2-5 |
| Nurse Session (n=4) | 4.33±0.96 | 5 | 2-5 |
| Dietitian sessions (n=6) | 4.59±0.64 | 5 | 3-5 |
| How would you rate your level of satisfaction with the length of the: |  |  |  |
| Health coaching sessions (n=6) | 4.74±0.53 | 5 | 3-5 |
| Nurse Session (n=4) | 4.74±0.66 | 5 | 2-5 |
| Dietitian sessions (n=6) | 4.67±0.55 | 5 | 3-5 |
| How would rate your level of satisfaction with the content of the materials covered by the: |  |  |  |
| Health coach) | 4.56±0.64 | 5 | 3-5 |
| Nurse | 4.48±0.75 | 5 | 2-5 |
| Dietitian | 4.59±0.63 | 5 | 3-5 |

Each of the questions were asked on a Likert scale of 1-5 (very satisfied, somewhat satisfied, neither, somewhat dissatisfied, dissatisfied; or very helpful, somewhat helpful, neither helpful or unhelpful, somewhat unhelpful, unhelpful).
